# The New York Institute of Stomatology

**Published:** 1900-03

**Authors:** 

**Affiliations:** The New York Institute of Stomatology


### THE NEW YORK INSTITUTE OF STOMATOLOGY.

A REGULAR meeting of the Institute was held on Thursday evening, November 9, 1899, at the office of Dr. J. Morgan Howe, No. 58 West Forty-seventh Street, New York, Dr. Charles O. Kimball, in the chair.

The minutes of the last meeting were read and approved.

*The Chairman.*—As there are no communications on theory and practice, we will proceed to the regular business of the evening. Inasmuch as Drs. Curtis and Lilienthal have not yet arrived, we will reverse the regular order of business. I will call on Dr. S. Freeman, who is to present a paper upon "The Use of Compressed Air in Operative Dentistry."

(For Dr. Freeman's paper, see page 162.)

*Dr. Freeman.*—I also wish to present a new antiseptic mouth-lamp. The lamp consists simply of an electric mouth-lamp, surrounded by a glass tube. The whole thing can easily be taken apart and cleansed. The double glass also has the advantage of taking up the heat from the lamp. The apparatus can be inserted in the nose, for instance, for several minutes without producing any appreciable warmth.

### DISCUSSION.

*Dr. John A. Schmidt.*—The paper was a very interesting one to me, inasmuch as I have used compressed air for several months, and find it a valuable adjunct to my office. The simple fact that I can use a spray, operated by my lady assistant, while cutting a tooth for crowning or while trimming down a filling, thus doing away with the pain of the operation due to thermal changes, more than pays for the apparatus. Again, before operating, spraying the mouth with an antiseptic bath makes the mouth pleasanter for the operator and is beneficial to the patient. Using the spray after removing the rubber dam leaves the mouth in a nice, fresh condition. For this purpose I use a modified form of listerine, which I find so satisfactory that I should like to give the formula to the Society.

℞ Benzoic acid,
Borax, āā ℥v;
Boric acid, ℥x.

Dissolve in *hot* water twelve pints.

℞ Ol. Eucalyptus,

Ol. Gaultheria,

Ol. Menth. pip., āā ʒii;

Thymol, ʒiv;

Fl. ext. wild indigo as coloring matter, ℥ii.

Dissolve in six pints of alcohol.

Mix altogether and add water sufficient to make four gallons. Allow to stand twelve hours and then filter. It is usually necessary to filter three times and obtain a beautiful straw-colored solution.

*Dr. Freeman.*—Do you cut the oils first with alcohol?

*Dr. Schmidt.*—Yes. This solution is too strong to use and must be diluted in one-half or more of water. For cases of pyorrhœa I use a one-half of one per cent. solution of formaldehyde, coloring this green.

Another nice spraying solution is one put upon the market recently by Johnson & Johnson, called "camphenol."

A five-ounce mixture costs thirty-five cents, and makes with the addition of water, four gallons of spraying-solution. Pyrozone or dioxide of hydrogen with borine or listerine also make good solutions.

We frequently find, in patients with a catarrhal affection, difficulty in breathing when the dam has been adjusted. In these cases I find the following solution very beneficial:

℞ Albolene, ℥ii;

Menthol, grs. v;

Ol. Eucalyptus, ♏xx.

M.—Use in an oil atomizer.

Close the lips firmly on the tube of the atomizer and spray, instructing the patient to inhale naturally and exhale through the nose. A patient suffering with coryza or any catarrhal condition is relieved and thus prepared for the dental operation, making it bearable. If you have not the compressed air and atomizer, any hand oil atomizer will give results which will pay for the trouble.

The method of heating the air for the hot-air syringe is of interest. The S. S. White heater in my hands has not been a success. I think the one Dr. Freeman describes ought to be successful. At present I am using cold air, as I have no heating apparatus. The apparatus which I have been using is made by the Cleveland Faucet Company. I have an apparatus in each operating room, in the laboratory for the blow-pipe, and also for inflating bicycle tires. I have a pump in the cellar, and the whole apparatus only cost me eighty dollars.

*Dr. Virgil F. Parker.*—I think I have contrived a little hot-air syringe which will suit Dr. Schmidt's purpose admirably. It consists of a coil of very small brass tubing surrounded by a jacket, the whole being filled with molten zinc. The current of air is made to pass through the coiled tube, and when the apparatus has been placed in the Bunsen or the alcohol flame for two or three minutes and becomes thoroughly heated the results are very satisfactory. It will give a continuous hot blast for ten or more minutes, which enables one to thoroughly dry a devitalized tooth to the extreme end of the roots, and I consider this of great importance towards the success of filling roots of teeth. If crowns are set with gutta-percha and for any reason require removal, it is possible to heat the crown sufficiently with this apparatus to soften the gutta-percha and easily slip the crown off.

*Dr. W. St. George Elliott.*—The lamp which Dr. Freeman has shown us seems to fill the requirements very fully. Of course, most of us know that we can buy an aseptic mouth-lamp at almost any electrical supply stores, made in the same way, only more crude, but which, however, is a very fair instrument.

In regard to the hot-air heaters, some one will introduce an instrument which answers the purpose fairly well. It will be taken up by the profession and used very largely until some one finds out that it is exceedingly inefficient. The reason for this is that many of our appliances are not tested for efficiency.

If you go into a manufacturing concern and inquire regarding any given machine, you will be told what commercial and theoretical efficiency the machine possesses. About a year ago I commenced a series of experiments to determine which was the most efficient. I took one milligram of water, and with the different apparatus heated for the same length of time in the same Bunsen flame, I evaporated that amount of water. The first thing that I ascertained was that the carbon which is put in so many of the heaters is not only useless, but is absolutely a detriment. I also found that in an absolutely new instrument no evaporation whatever took place. This was due to the fact that the paraffin which covers the carbon when it is new, melts and forms a covering over the water, preventing evaporation. I found that the most efficient heater is the Perry, which is a little cone of copper or silver at the end of a syringe. I also experimented with long tubes of brass, one-sixteenth of an inch in diameter and about fifteen feet long. These were formed into a small coil and placed in a tank of boiling water. Passing a stream of air through this tube I found that the results amounted to nothing. The air passed through the tube so rapidly that it absorbed practically no heat whatsoever. I am now experimenting a little with a view to using the gas blow-pipe itself. An excellent method of introducing medicinal vapors into root canals is by heating an ordinary syringe in the flame and then drawing a drop of the medicament into the syringe and returning it as vapor.

I have also made an electrical apparatus for pumping the air, the hydraulic apparatus being very apt to break down.

I have a little fan motor behind my cabinet, on the floor, which runs a grinding and polishing apparatus, and the same motor runs a fan in summer. I connected with this apparatus a small bicycle pump, which pumps air into a reservoir containing ten gallons. This gives me a pressure of ten pounds, which I find is sufficient.

*Dr. Freeman.*—If Dr. Elliott will go to the Watler Manufacturing Company, he will find the very apparatus which he has described. This apparatus will give fifteen pounds pressure, the cylinder holding ten or fifteen gallons. It may also be used as a suction-pump by reversing.

*Dr. Charles Meeker.*—I would like to ask Dr. Freeman what brand of hydrogen dioxide he uses and also if he has used hydrozone.

*Dr. Freeman.*—The hydrogen dioxide which I use is that prepared by the Oakland Chemical Company. I have never used the hydrozone.

*The Chairman.*—We will now bring the discussion to a close and with thanks to the gentlemen who have so kindly participated. Dr. C. B. Parker, much to our regret, is unable to be with us this evening. He has, however, sent on his paper, and, with your permission, the secretary will read it. I regret exceedingly his absence, as we had hoped to have Dr. Parker's reply to the discussion as well as his paper.

(For Dr. Parker's paper, see page 169.)

DISCUSSION.

*G. Lenox Curtis.*—An apology seems unnecessary by one able to do conservative surgery as described by Dr. Parker.

My experience in the use of cocaine is confined to hydrochlorate. This I use in varying strength, to suit the case.

I invariably employ its antidote,—volasem,—to guard against any untoward effects. This enables me to proceed with the operation without loss of time, and the patient to leave my office as soon as the operation is over. I use cocaine in fully ninety per cent. of my operations.

The dentist's manual training, and familiarity with oral work, enables him to operate within the mouth with the bur and engine as readily as the general surgeon operates with the knife and cutting forceps from without. The great advantage of this, as claimed by the author, is the little damage done to the facial expression, for reason of no scarring, small loss of blood, and little injury to the nerves. It also facilitates rapid operating, and, because of the minimum amount of injury to the normal tissues, the time required in the reproductive process is greatly reduced.

If surgeons would familiarize themselves with the use of the engine and this method of operating, I think they would find it a great advantage. I believe surgeons are not justified in cutting through the face to do maxillary operations. Operations through the mouth have been demonstrated for so long a time that every up-to-date surgeon must recognize them. It would, therefore, seem that it is the duty of the surgeon, in event of unfamiliarity with his work, to call in a man like Dr. Parker to assist him. It is now considered no disgrace to admit we do not know everything.

It also seems unjustifiable not to teach the student these conservative methods. Nothing to me seems more crude in surgery than to cut through the face in maxillary operations, such as in the disease of the antrum and resecting the maxilla; and yet how few surgeons there are who consider personal appearance in this work. In Germany it is considered an honor to display facial scars; in America, a misfortune.

I can understand some of the advantages derived from the use of the lead plate, for instance, the retaining of the contour of the face until sufficient bone is formed to support the periosteum, and also checking granulation when complete. However, this method is new to me.

In my article on "Resection and Reproduction of the Maxillæ," published in the INTERNATIONAL DENTAL JOURNAL of March 19, 1898, I speak of the preservation of the contour of the face, from which I quote the following:

"My method to obtain the best results in the preservation of the contour of the jaw is by retaining the necrosed bone in position until the periosteum has been so strengthened by reproduction as to allow nature's outlines to be maintained, employing it as an interosseous splint. Where it is necessary to remove the bone, I retain the contour of the face by gauze packing, and change from time to time until the bone is sufficiently reproduced to resume its shape.

"Where the destruction of the bone has been great, and the periosteum too weak to retain the jaw in position during the process of reproduction, I use an interdental splint (as employed by Liston over fifty years ago), in which the upper and lower teeth properly occlude."

This method has been employed by me for a period extending over more than a dozen years.

*The Chairman.*—As this is a subject which requires more of a surgical knowledge, I have asked Dr. H. Lilienthal, of Mount Sinai Hospital, to be present and discuss Dr. Parker's paper.

*Dr. H. Lilienthal.*—I have listened with great interest to this paper, which I think is a decidedly important one, since it deals with a class of cases which, as Dr. Curtis has said, certainly have been neglected by the general surgeon. I will admit that, until comparatively recent years, but little attention has been paid to the sometimes frightful disfigurement which follows the removal of the superior maxillary bones.

In the first place as to anæsthesia. I do not think it is exactly fair, although I understand Dr. Curtis's feeling in the matter and do not wish to go outside of Dr. Parker's paper, to judge of the possibilities of local anæsthesia simply from cases of caries or necrosis, for that is only one of the reasons for the removal of the superior maxillary bones. Nature has really removed them, or will do so if we wait long enough, although of course with unsightly deformities.

We must also recognize the fact that when we are not dealing with caries and necrosis, but with neoplasms, it may be necessary to work in a more radical fashion than is done in the beautiful method described by Dr. Parker. For instance, in sarcoma of the superior maxilla, where we have to take away bone, periosteum, nerves, and in some instances the skin as well.

Of course, these are not the cases to which Dr. Parker has referred; nevertheless, I refer to them because I do not think it exactly right that a stomatologist who sees a case of disease of the superior maxilla should immediately jump at the conclusion that the operation is comparatively trifling and that local anæsthesia will suffice. Of course, in cases of caries and necrosis this may be so. It is, therefore, very important to diagnose the case very carefully in the beginning to ascertain whether you are really dealing with one of the cases which have been referred to, or whether it is perhaps neoplastic in origin.

I have done considerable general surgery with the aid of local anæsthetics. I have opened the gall-bladder a number of times and removed gallstones. Opened the urinary bladder. Done many operations for hernia. Only recently I resected a costal cartilage and opened the pericardium, removing from fifty to sixty ounces of pus. The patient made a recovery and the operation was done with absolutely no pain. In this case it would have been exceedingly dangerous to have employed a general anæsthesia.

I believe in local anæsthesia, but I think in a resection of the upper jaw, where you have more to do than the removal of a sequestrum or the scraping of a carious cavity, where you have to cut through much soft tissue, as in removing the entire superior maxilla, exposing the eye and orbit, the operation is so horrible that I think a general anæsthetic is absolutely indispensable.

As to local anæsthetics I will make one more remark : There are two drugs besides cocaine which are valuable. They are beta eucain and nirvanin, which latter is a soluble form of orthoform.

The bur was mentioned as being preferable to the chisel in these operations. The reason why the surgeon sticks to the chisel rather than to the forceps or saw is that the chisel makes a clean cut, so where it is necessary that the bone should heal without giving off the smallest quantity even of necrotic tissue, the chisel is the only instrument which is suitable. In the operations described, of course, it does not make so much difference; nevertheless, the use of the chisel should not be forgotten.

The problem of keeping the facial expression anything like what it normally was, is a very complicated one, particularly where the periosteum has been removed and there is no regeneration of bone. There I think the highest skill of the stomatologist comes into play, and I recognize the fact that every man must have his own method. I do not believe any two cases are alike. The tissue in no two instances is alike. I have seen some very beautiful and some very mediocre attempts in this direction. I call attention to the frightful deformity when both superior maxillæ are removed. The difficulties in these cases must be fully ten times as great as when but one side is taken away. I have seen many cases where one superior maxilla has been removed and the facial expression was very good. I have seen only one or two where both have been removed and none where the deformity was not great.

The lead method, as described by Dr. Parker, is very clever; this splint keeping the granulation in place until healing is well under way. The only difficulty would be where we have no regeneration of bone. Here the cicatricial contraction will continue long after the gauze has been removed. However, this method would suffice in the cases covered by the paper, but some method should be invented for keeping the soft parts up without the use of gauze, which is compressible and allows the deformity to progress. I will say that the only good I have ever seen from an appliance in cases of double resection of the superior maxilla was in the separation of the nasal from the oral cavity. Of course, when both maxillæ are gone the mouth and nose form one huge cavity. An artificial roof of the mouth, here, which will separate the nose from the mouth, will contribute enormously to the patient's health and comfort. I have seen such an appliance made by a dentist. The patient protested because the face was not as beautiful as before the operation, but inasmuch as the dentist did not see her until the cicatricial tissue had formed, he was not to be blamed.

*The Chairman.*—We are very much obliged to our friend, Dr. Lilienthal. We are disappointed this evening in the absence of Professor Dawbarn, who was to have been present and take part in the discussion, but he informed me that he had overlooked the fact, when he promised to be present, that he had an important faculty meeting for this evening which he had to attend.

There is present with us this evening Dr. Fuller, of Brooklyn, who has seen some of Dr. Parker's operations, and could perhaps tell us further about them.

*Dr. D. A. Fuller.*—I have seen two or three of the operations, but I do not know that I can say anything more than has been said. I was very much pleased indeed with them, and think them equal to any operations in oral surgery which I have ever seen. I saw the operation, the results of which are shown in the picture, and was very much gratified with everything which occurred in connection with the operation.

*The Chairman.*—Gentlemen, with your permission, I will endeavor to explain, as Dr. Parker told me, the manner of application of the lead a little more in detail. In applying the lead splint, it is desirable to get some support from the back teeth, and, as Dr. Lilienthal has pointed out, the special advantage of Dr. Parker's method is in just these cases where the whole of the two maxillæ have not been removed, but where the anterior portion has been removed. Dr. Parker selects some small hollow or pit in the bone left on either side, and to these adjusts a little point of the lead splint; if there be no good catch he makes one. The lead plate is then placed inside the lips, the little points caught in the pits of bone, and the natural elasticity of the lips holds it in place without any further adjustment. The lead is in sheets, about the thickness of thin pasteboard, thick enough to hold, but thin enough so that it is in position, contouring the face as desired. The appliance is then removed and wrapped with two thicknesses of gauze and replaced in position, and as the granulations approach it at the upper edge it is cut away, thus holding out the lips and face during granulation.

*Dr. Charles A. Meeker.*—I should like to have Dr. Lilienthal explain somewhat more fully about nirvanin. What per cent. solution is used as a local anæsthetic?

*Dr. Lilienthal.*—I have used nirvanin anywhere from one-half to four per cent. I generally use a two per cent. solution. It is injected hypodermically. The special advantage is that the anæsthesia lasts longer. It does not last so long as orthoform, but longer than cocaine or eucain. With eucain (not beta) I have had one or two disagreeable experiences. One, a case of circumcision, where I did not want to give a general anæsthetic, I injected eucain (not beta) with very disastrous results, necrosis of the skin occurring. With the beta eucain, however, I have never had such an experience, as I have no doubt that if I had used it in this instance the result would have been different. Beta eucain was not then on the market.

*Dr. Curtis.*—In resection of the upper maxillæ in consequence of necrosis, I would say if an impression of the upper jaw is taken before the operation, a cast run, and an interdental splint made to hold the teeth in position during the process of reproduction, the bone will become attached to the teeth and the teeth will become as useful as ever. The teeth are thus retained in normal position and the contour of the face remains unchanged. This method is also applicable to the lower jaw.

*Dr. Lilienthal.*—I was referring to cases where there was complete resection of the superior maxillæ. Of course, if there are no teeth we cannot make an inderdental splint. I do believe Dr. Curtis is correct where there are any teeth, and he is wise in having his prosthetic appliance made beforehand.

*The Chairman.*—It is very unfortunate that our paper of the evening was read instead of being presented by Dr. Parker in person, because there are many things upon which he would have been able to enlighten us in the discussion. If there is nothing further to be said on the subject, we will close the discussion with our thanks to the gentlemen who have come among us and addressed us so ably and scientifically.

Adjourned.

FRED. L. BOGUE, M.D., D.D.S.,
*Editor the New York Institute of Stomatology.*

The annual meeting of the Institute was held on Tuesday evening, December 5, 1899, at the office of Dr. S. E. Davenport, No. 51 West Forty-seventh Street, New York, Dr. Charles C. Kimball in the chair.

The following officers were elected for the year 1900.

*President*, E. A. Bogue. *Vice-President*, C. A. Woodward. *Recording Secretary*, F. Milton Smith. *Corresponding Secretary*, George A. Wilson. *Treasurer*, J. A. Bishop. *Editor*, F. L. Bogue. *Curator*, J. C. Palmer. *Executive Committee*, J. Morgan Howe, C. O. Kimball, S. E. Davenport.

